# Academic Life in Emergency Medicine (ALiEM) Blog and Podcast Watch: Gastrointestinal Emergencies

**DOI:** 10.7759/cureus.5545

**Published:** 2019-09-01

**Authors:** Jay Khadpe, Eric J Morley, Salim R Rezaie, Andrew Grock

**Affiliations:** 1 Emergency Medicine, University of Florida College of Medicine, Jacksonville, USA; 2 Emergency Medicine, Stony Brook Medicine, New York, USA; 3 Emergency Medicine, Methodist Hospital, San Antonio, USA; 4 Emergency Medicine, University of California, Los Angeles, USA

**Keywords:** emergency medicine, free open access medical education, gastrointestinal emergencies

## Abstract

The Academic Life in Emergency Medicine (ALiEM) Approved Instructional Resources (AIR) Series and Approved Instructional Resources - Professional (AIR-Pro) Series were created in 2014 and 2015, respectively, in response to the growing need to curate online educational content as well as create a nationally available curriculum that meets individualized interactive instruction criteria for emergency medicine (EM) trainees. These two online series identify high-quality educational blog and podcast content using an expert-based approach. We summarize the accredited posts on gastrointestinal emergencies that met our a priori determined quality criteria per evaluation by eight experienced faculty educators in EM.

## Introduction and background

Emergency medicine (EM) has been at the forefront in the adoption of social media platforms as a means to disseminate knowledge with a rapid rise of educational content available through blogs and podcasts [1]. However, the assessment of quality with regard to these online educational resources for both educators and learners has only made preliminary progress [2-4]. Additionally, in 2008, the Accreditation Council for Graduate Medical Education endorsed a decrease in synchronous conference experiences for EM residency programs by up to 20% in exchange for what was termed Individualized Interactive Instruction (III) [5]. Therefore, a need emerged to develop a national curriculum that both satisfied requirements for III as well as established a standardized method to assess quality for online educational resources. The Academic Life in Emergency Medicine (ALiEM) Approved Instructional Resources (AIR) Series and Approved Instructional Resources - Professional (AIR-Pro) Series were created in 2014 and 2015, respectively, to fill these gaps [6-7]. Using an expert-based, crowd-sourced approach, these two programs identify high-quality educational blog and podcast content. The ALiEM Blog and Podcast Watch series is authored by members of the AIR and AIR-Pro Series’ editorial boards to create written summaries of these selected posts [8-17]. This installment from the series summarizes the highest-scoring social media educational resources on gastrointestinal emergencies.

## Review

Topic identification

The AIR Series is a continuously building curriculum structured to cover all relevant organ systems.

Inclusion and exclusion criteria

A search of the 50 most frequently visited sites per the Social Media Index was conducted for resources relevant to gastrointestinal emergencies, published within the previous 12 months [18-19]. The search, conducted in September 2017, included blog posts and podcasts written in English for scoring by our expert panel.

Scoring

Extracted posts were scored without blinding by eight reviewers from the AIR Editorial Board, which is comprised of EM core faculty from various United States medical institutions. The scoring instrument contains five measurement outcomes using seven-point scales: Best Evidence in Emergency Medicine (BEEM) score, accuracy, educational utility, evidence-based medicine, and references (Table 1) [20]. More detailed methods are described in the original description of the AIR Series [6-7]. Board members with any role in the production of a reviewed resource recuse him/herself from grading that resource.

**Table 1:** Approved Instructional Resources scoring instrument for blogs and podcasts. BEEM: Best evidence in emergency medicine; EP: Emergency physician; EBM: Evidence-based medicine.

Tier 1: BEEM Rater Scale	Score	Tier 2: Content accuracy	Score	Tier 3: Educational Utility	Score	Tier 4: Evidence Based Medicine	Score	Tier 5: Referenced	Score
Assuming that the results of this article are valid, how much does this article impact on EM clinical practice?		Do you have any concerns about the accuracy of the data presented or conclusions of this article?		Are there useful educational pearls in this article for senior residents?		Does this article reflect evidence based medicine (EBM)?		Are the authors and literature clearly cited?	
Useless information	1	Yes, many concerns from many inaccuracies	1	Not required knowledge for a competent EP	1	Not EBM based, only expert opinion	1	No	1
Not really interesting, not really new, changes nothing	2		2		2		2		2
Interesting and new, but doesn't change practice	3	Yes, a major concern about few inaccuracies	3	Yes, but there are only a few (1-2) educational pearls that will make the EP a better practitioner to know or multiple (>=3) educational pearls that are interesting or potentially useful, but rarely required or helpful for the daily practice of an EP.	3	Minimally EBM based	3		3
Interesting and new, has the potential to change practice	4		4		4		4	Yes, authors and general references are listed (but no in-line references)	4
New and important: this would probably change practice for some EPs	5	Minimal concerns over minor inaccuracies	5	Yes, there are several (>=3) educational pearls that will make the EP a better practitioner to know, or a few (1-2) every competent EP must know in their practice	5	Mostly EBM based	5		5
New and important: this would change practice for most EPs	6		6		6		6		6
This is a "must know" for EPs	7	No concerns over inaccuracies	7	Yes, there are multiple educational pearls that every competent EP must know in their practice	7	Yes exclusively EBM based	7	Yes, authors and in-line references are provided	7

Resources with a mean evaluator score of ≥ 30 points (out of a maximum of 35) are awarded the AIR label. Resources with a mean score of 27-29 and deemed accurate and educationally valuable by the reviewers are given the Honorable Mention (HM) label.

Results

We initially included a total of 78 blog posts and podcasts. Key educational pearls from the one AIR post and the seven HMs are described.

Article 1. Pescatore R. Should You Give Albumin in Spontaneous Bacterial Peritonitis (SBP)? RebelEM. (June 5, 2017) AIR

http://rebelem.com/should-you-give-albumin-in-spontaneous-bacterial-peritonitis-sbp/ This blog post provides an evidence-based analysis of the benefit of albumin in the treatment of SBP.

Take-home points: The authors identified two randomized controlled trials evaluating intravenous (IV) albumin for SBP [21-22]. Both trials took place in the university hospital setting comparing IV antibiotics plus albumin infusion to IV antibiotics alone and found a statistically significant reduction in rates of renal failure and in-hospital mortality in the intervention group. Additionally in 2012, the American Association for the Study of Liver Diseases published guidelines recommending antibiotics and IV albumin (1.5 mg/kg) within six hours of diagnosis for patients with SBP, as defined by an ascitic fluid polymorphonuclear leukocyte count greater than 250 cells/mm^3^ with clinical suspicion for SBP, as well as any of the following: serum creatinine > 1 mg/dl, blood urea nitrogen (BUN) > 30 mg/dl, or total bilirubin > 4 mg/dl. The author of this post agrees that the evidence supports timely administration of albumin to emergency department (ED) patients diagnosed with SBP and with either elevation in creatinine, BUN, or total bilirubin [23].

Article 2. Rezaie S. The Good, The Bad, and The Ugly of Proton Pump Inhibitors in UGIB. RebelEM. (February 23, 2017) HM

http://rebelem.com/good-bad-ugly-proton-pump-inhibitors-ugib/

This blog post reviews literature for the use of proton pump inhibitors (PPIs) in upper gastrointestinal bleeding (UGIB).

Take-home points: Three meta-analyses were reviewed that address three different questions: do PPIs benefit patients with known peptic ulcer disease (PUD) and UGIB, do PPIs benefit patients with undifferentiated UGIB, and is a PPI infusion in addition to a bolus dose more effective than a bolus dose alone [24-26]? They report that PPIs reduce the need for surgical intervention and rebleeding, but do not decrease mortality in patients with known PUD and UGIB. PPIs have no effect on mortality, rebleeding rate, or need for surgical intervention in patients with undifferentiated UGIB, but there is a reduction in the stigmata of recent bleeding and need for endovascular therapy. Adding an infusion to the bolus dose has no effect on rebleeding rate, mortality, number of transfusions, or hospital length of stay [27].

Article 3. Fisher R and Milne K. SGEM#180: The First Cut is the Deepest - N.O.T. for Paediatric Appendicitis. The Skeptics’ Guide to EM. (May 24, 2017) HM

http://thesgem.com/2017/05/sgem180-the-first-cut-is-the-deepest-n-o-t-for-paediatric-appendicitis/

This blog post/podcast provides a critical appraisal of a meta-analysis done by Georgiou et al. on the efficacy and safety of non-operative treatment of pediatric appendicitis (NOTA) [28]. 

Take-home points:​​​​​​​​​​​​​ Acute uncomplicated appendicitis is traditionally treated with surgery, but there is an increasing number of publications demonstrating the safety of IV antibiotics alone without surgery. The reviewed study randomized 413 acute uncomplicated appendicitis patients to NOTA. Of note, four percent of patients (17/413) failed non-operative treatment and required surgery during the primary admission while the remaining 96% of patients (396/413) were safely discharged. While no adverse effects from NOTA were reported, 14% of patients discharged (68/396) had recurrent appendicitis at follow up. The podcast’s bottom line asserts that NOTA is an interesting management option for discussion, but not ready for widespread implementation [29].

Article 4. Harty S. Annals of B-Pod: Abdominal Compartment Syndrome. Taming the SRU. (June 22, 2017) HM

http://www.tamingthesru.com/blog/annals-of-b-pod/june-2017/abdominal-compartment-syndrome

This blog post reviews the etiologies, definition, identification, and management considerations of patients with abdominal compartment syndrome (ACS).

Take-home points:​​​​​​​​​​​​​​ ACS is a rare but deadly and under-diagnosed clinical condition that is defined as an intra-abdominal pressure (IAP) > 20 mmHg with associated end-organ damage. Identifying patients with ACS is challenging as they often are critically ill upon presentation. They may complain of abdominal pain, bloating, difficulty breathing, and lightheadedness. On physical exam, patients will have a distended abdomen that is tender and may have signs of peritonitis. Signs of shock may also be present such as tachycardia, hypotension, decreased urine output, and lactic acidosis. ACS can be classified as primary, due to trauma or a direct medical or surgical etiology, or secondary, due to iatrogenic aggressive fluid resuscitation typically in cases of sepsis or burns. In cases of ACS, the increased pressure reduces perfusion to the abdominal viscera leading to progressive ischemia, infarction, and perforation often with significant concomitant lactic acidosis. IAP can be measured using specialized transducer instruments through a nasogastric tube, rectal tube, or Foley catheter. In the absence of these specialized transducers, measurement of IAP can be achieved by connecting the arterial line pressure transducing tubing to a Foley catheter. Though the definitive treatment is surgical intervention, ACS can be initially treated by limiting fluid administration, decompressing the stomach and colon with nasogastric and/or rectal tubes, and performing paracentesis if abdominal ascites is present. Empiric antibiotic coverage for abdominal flora may be warranted as bacterial translocation occurs due to impaired perfusion to the intestines [30].

Article 5. Gray C. Upper Gastro Intestinal Bleeding at St.Emlyn’s. St. Emlyn’s. (September 17, 2016) HM

http://stemlynsblog.org/upper-gastro-intestinal-bleeding-at-st-emlyns/

This blog post and podcast comprehensively reviews the etiology, assessment, and resuscitation of UGIB.

Take-home points:​​​​​​​​​​​​​​ Though the majority of UGIB stem from PUD, variceal bleeds should be considered in patients with stigmata of cirrhosis. Patients often present with acute hemorrhage symptoms and signs such as dizziness, syncope, hypotension, and tachycardia. Patients may also present with frank bloody vomitus or may have more subtle signs of bleeding such as coffee ground emesis, dark vomitus, or dark stools. The Glasgow-Blatchford score may help risk-stratify patients with UGIB. Patients with a score of zero and who appear well may be discharged with a plan for outpatient endoscopy. Severely ill patients require immediate resuscitation to restore hemodynamic stability prior to endoscopy including large-bore IV catheters, fluids and/or blood transfusions, and often endotracheal intubation. Blood should be transfused to a target hemoglobin of 7 g/dL (9 g/dL in patients with unstable coronary artery disease). Platelets and fresh frozen plasma should be transfused if the platelet count is less than 50 x 109/L or the international normalized ratio is > 1.5. A balloon tamponade device (Sengstaken-Blakemore or Minnesota tube) should be placed if the patient cannot be stabilized. All patients require emergent endoscopy when sufficiently stable. Consider involving gastroenterology, critical care, and anesthesia consultants early in the presentation. Vasopressin and antibiotics should be administered in suspected or confirmed variceal bleeds [31].

Article 6. Weingart S. Podcast 196 - Having a Vomit SALAD with Dr. Jim DuCanto - Airway Management Techniques during Massive Regurgitation, Emesis, or Bleeding. EmCrit. (April 3, 2017) HM

https://emcrit.org/emcrit/having-a-vomit-salad-with-ducanto/

This blog post and podcast summarizes airway management techniques during massive regurgitation, emesis, or bleeding, specifically focusing on suction-assisted laryngoscopy and airway decontamination (SALAD). 

Take-home points:​​​​​​​​​​​​​​ One of the most difficult airway scenarios involves massive airway contamination. Options for managing this scenario include deliberate intubation of the esophagus with the endotracheal tube and gentle inflation of the balloon or placing the tip of a suction catheter in the esophagus. However traditional Yankauer suction catheters can become obstructed in the presence of solid debris. The DuCanto suction catheter, on the other hand, has a larger internal diameter that allows passage of solid debris (0.26-inch aperture opening).

The DuCanto catheter is shaped to work with hyperangulated laryngoscope blades and lacks a ventilation hole. It is held using an overhand grip which can help the catheter depress the mandible to assist in the placement of the laryngoscope (i.e., like a tongue depressor).

An important technique, the SALAD-Park maneuver, involves placing and then leaving the suction catheter in the esophagus prior to endotracheal intubation. The suction catheter should be on the left-hand side of the laryngoscope blade so that there is enough room in the oropharynx to intubate with an endotracheal tube.

Finally, the bougie can be passed directly through the DuCanto catheter to assist with bougie-guided intubation. However, this is an off-label use of the device and is currently under study [32].

Article 7. Faust J and Westafer L. Episode 73 - Gastroparesis & Biliary Pathology. FOAMcast. (September 1, 2017) HM​​​​​​​

http://foamcast.org/2017/09/01/episode-73-gastroparesis-biliary-pathology/

This blog post and podcast reviews both the evidence for haloperidol in treating gastroparesis as well as biliary pathology core content.

Take-home points: Ramirez et al. conducted a before and after study comparing patients who were given 5 mg haloperidol intramuscularly to a prior visit when they did not receive haloperidol [33]. The authors found a significant reduction in hospital admission and morphine equivalent administration in the haloperidol group but no difference in ED length of stay. The study’s conclusions are severely limited by its study design and small sample size of 52 patients.

In the study by Roldan et al., the authors conducted a randomized controlled trial comparing 5 mg of IV haloperidol to placebo in ED patients presenting with gastroparesis [34]. The study found a significant reduction in pain and nausea scores in the haloperidol group compared to the placebo group. The authors’ conclusions are primarily limited by the extremely small sample size of only 33 patients.

The core content section reviews the approach to biliary pathology diagnosis in the ED. For diagnosing acute cholecystitis, clinician gestalt has a +LR of 25-30. Labs may be variable, and while the hepatobiliary scan is the most sensitive imaging test, ultrasound (US) is the preferred ED imaging modality. Cholecystitis treatment includes antibiotics, source control (cholecystectomy or cholecystectomy tube), and symptom control. Cholangitis is characterized by Charcot’s triad of fever, right upper quadrant abdominal pain, and jaundice or Reynold’s pentad which adds hypotension and altered mental status. US or computed tomography (CT) scan can aid in the diagnosis and treatment includes antibiotics, source control, often endoscopic retrograde cholangiopancreatography for stone removal, and symptom therapy [35].

Article 8. Rezaie S. Question Tradition: Glucagon for Food Boluses. RebelEM. (January 2, 2017) HM​​​​​​​​​​​​​​

http://rebelem.com/question-tradition-glucagon-food-boluses/

In this blog post, the authors performed an evidence-based review of the efficacy of glucagon for the treatment of esophageal food bolus obstructions.

Take-home points: Endoscopy is the preferred treatment for esophageal food bolus obstructions. However, glucagon has been described as a potentially helpful medication that could avoid this procedure. The authors of this blog post performed a literature search on the efficacy of glucagon for relieving food bolus obstructions as well as any side effects. They found eight trials that met their inclusion criteria, most of which were small and low quality. Three of the studies were placebo-controlled [36-38]. In these three studies, glucagon relieved the obstruction in 9.45 - 37.5% of patients compared to 10.3% - 31.6% of patients who received a placebo. Additionally, 10.6% of patients had nausea and vomiting and 22.3% had esophageal pathology. The authors concluded that glucagon is not effective for esophageal food bolus obstructions and endoscopy should be the first-line treatment modality [39].

## Conclusions

The ALiEM Blog and Podcast Watch series identifies high-quality educational blogs and podcasts for EM clinicians through its expert panel, using an objective scoring instrument. These social media resources are currently curated in the ALiEM AIR Series, originally created to address EM residency needs. While this article focuses on gastrointestinal emergencies, additional AIR modules address other topics in EM. The resources chosen specifically for gastrointestinal diseases are shared and summarized here, to help clinicians and educators filter through the rapidly published multitude of blog posts and podcasts. Our search was limited to content produced within the previous 12 months from the top 50 Social Media Index sites. While these lists are by no means a comprehensive analysis of the entire internet for this topic, the AIR and AIR-Pro series provide post-publication accreditation and curation of recent online content to identify and recommend high-quality, educational, social media content for the EM clinician.
